# Supplementary material for: Widespread fungal–bacterial competition for magnesium lowers bacterial susceptibility to polymyxin antibiotics
Source: PLoS Biol. 2024 Jun 20;22(6):e3002694. doi: 10.1371/journal.pbio.3002694 (PMC11218974; doi:10.1371/journal.pbio.3002694)
Supplement: Attachment — Submitted filename: 143272_1_rebuttal_2982440_sb5gnf.docx [file pbio.3002694.s044.docx]

**Overall comments**

We are pleased by the reviewers’ comments and appreciate their suggestions for improvements. In addition to correcting small typos throughout the manuscript, we have made the following additions or changes in response to reviewer comments and suggestions:

1. New complementation experiments to verify the impacts of *mgtA* and *PA4824* on bacterial fitness in fungal co-culture.
2. New experiments to measure intracellular Mg^2+^ levels in *corA* or *mgtE* mutants to strengthen our conclusion that neither of these constitutive Mg^2+^ transporters is required for maintaining intracellular Mg^2+^ levels in co-culture.
3. New experiments to confirm that the *S. cerevisiae* *mnr2*Δ mutant does not have a fitness defect compared to WT in co-culture. This finding rules out the possibility that metabolic defects in the *mnr2*Δ mutant restore the fitness of bacterial *mgtA* mutant in co-culture and strengthens our hypothesis that Mg^2+^ sequestration by fungal vacuole triggers Mg^2+^ nutritional competition with bacteria.
4. Clarification of bacterial species we tested in our study as suggested by Reviewer #3.
5. Revised discussion to highlight how our findings relate to any fungal-bacterial interaction both in ecological and infection contexts and any known role of *mgtA* in antibiotic susceptibility, as suggested by Reviewer #2.

All changes in response to the reviewer’s comments have been detailed in our point-by-point response (below).

**Reviewer #1** (Evidence, reproducibility and clarity (Required)):

This manuscript investigates polymicrobial interactions between two clinically relevant species, Pseudomonas aeruginosa and Candida albicans. The findings that C. albicans mediates P. aeruginosa tolerance to antibiotics through sequestration of magnesium provides insight into a specific interaction at play between these two organisms, and the underlying mechanism. The manuscript is well composed and generally the claims throughout are supported by the provided evidence. As a result, most comments are either for clarification, or minor in nature.

We thank the reviewer for their positive comments and their suggestions for improvement.

Major comments:

1) For their experiments, the authors frequently switch between 30C and 37C, but there is no rationale for why a specific temperature was used, or both were. E.g. some of the antibiotic survival assays, and fungal-bacterial co-culture assays were performed at both temperatures, while the colistin resistance, fitness competition and RNA sequencing were performed at 30C. Given the fact that the two organisms are both human pathogens and co-exist in human infections, it is not clear why 30C was used. The authors should provide clarity for why these two temperatures were used.

We thank the reviewer for raising this point. Fungal-bacterial interactions occur in a range of temperatures in ecological contexts (*e.g.,* in soil or on plants) or during infection in animal hosts. Both 30^o^C and 37^o^C degree temperatures are used in *C. albicans* studies whereas 37^o^C is most preferred for *P. aeruginosa* studies. By providing data from both temperatures for critical experiments, we demonstrate that our findings are not dependent on temperature. Our studies also allow for an easy comparison to previously published studies performed at both temperatures. We chose to screen initial co-culture conditions showing fungal antagonism at 30^o^C, as *C. albicans* cells can reach higher CFUs than at 37^o^C due to growth in the single-celled yeast form.

We agree with the reviewer that 37^o^C is more physiologically relevant for conditions under which these two species coexist in animal hosts. Thus, we tested our findings of Mg^2+^ competition and antibiotic survival at 37^o^C.

We now clarify our reasoning in the revised Materials and Methods section as follows: “*We chose 30^o^C for the initial co-culture assays for two reasons. First, C. albicans cells reached higher CFU at 30^o^C than 37^o^C, which would impose a stronger competition with bacteria. Second, C. albicans cells form hyphae at 37^o^C, which can have multiple cells in one filament and thus confound CFU measurements. We further confirmed that our findings of Mg^2+^ competition are independent of temperatures by setting up co-culture assays at both 30^o^C and 37^o^C.*”

2) Lines 184-191: It would be useful to measure intracellular Mg2+ (using the Mg sensor) in the corA and mgtB tn mutants in media as well as the fungal spent media, to provide stronger support for the claim that "MgtA is a key bacterial Mg2+ transporter that is highly induced under low Mg2+ conditions".
We thank the reviewer for this suggestion. Based on our experiments, neither CorA or MgtE are induced (in RNA-seq analyses) nor required in co-culture (in Tn-seq analyses), suggesting neither is involved in Mg^2+^ competition with *C. albicans*. In contrast, MgtA is highly induced in co-culture. Loss of *mgtA* significantly reduces bacterial fitness in co-culture and intracellular Mg^2+^ levels only in *C. albicans*-spent BHI, but not fresh BHI. These results suggest that MgtA is the key Mg^2+^ transporter required for bacterial Mg^2+^ uptake and fitness in co-culture.

Nevertheless, we agree with the reviewer that despite being constitutively expressed, CorA or MgtE might play an important role in importing Mg^2+^ in BHI and *C. albicans*-spent BHI. To test this possibility, we performed a new experiment suggested by the reviewer (now included in the revised manuscript) in which we measured intracellular Mg^2+^ levels in *corA* or mgtE loss-of-function mutants in BHI versus *C. albicans*-spent BHI, and compared them to intracellular Mg^2+^ levels in a *mgtA* loss-of-function mutant strain. We find that lack of either *corA* or *mgtE* does not significantly reduce bacterial Mg^2+^ levels in *C. albicans*-spent BHI compared to Δ*mgtA* mutant (Fig. S7C). Thus, our results strengthen our conclusion that MgtA is the key Mg^2+^ transporter that gram-negative bacteria use to overcome fungal-mediated Mg^2+^ sequestration.

3) Line no. 276. Does the mnr2∆ S. cerevisiae mutant have a growth defect compared to the WT? This would test whether the effect of the mnr2 mutant on P. aeruginosa fitness is strictly due to Mg2 and not due to reduced growth or metabolism of the mutant.
We agree with the possibility raised by the reviewer. In new experiments included with our revision as Figure S10, we find that the *S. cerevisiae* mnr2 deletion mutant exhibits similar CFU as WT in monoculture as well as co-culture. Thus, the rescuing effect of *mnr2*Δ is less likely due to reduced growth or metabolism.

4) The authors use the term 'antibiotic resistance' throughout the manuscript. However, the assays they perform do not directly test for antibiotic resistance which is defined as the ability to grow at higher concentrations of antibiotics (e.g. as measured by MIC tests). The authors should rephrase their phenotype as antibiotic survival or antibiotic tolerance.

We agree with the reviewer and thank them for raising this point. We replaced the phrase ‘antibiotic resistance’ with ‘antibiotic survival’ throughout the revised manuscript. We also accordingly changed our title to ‘Widespread fungal-bacterial competition for magnesium lowers antibiotic susceptibility’

5) Also, the authors have two different assays, both measuring survival in antibiotic, but one is called a colistin resistance assay (line 508) and the other a colistin survival assay (line 523). It's not obvious what is the difference between what is being assayed in the two experiments, except perhaps the growth phase of the cells when they are exposed to the antibiotic? The authors should explain the difference, and the rationale for using two different assays.

We thank the reviewer for raising this point. In the revised manuscript, we explain the rationale of our two assays. The first assay measures the bacterial survival after colistin treatment in *C. albicans*-spent BHI, and the second measures the bacterial survival after colistin treatment in co-culture with *C. albicans*. We performed both assays because *C. albicans*-spent BHI mimics Mg^2+^-depleted conditions by *C. albicans* but might not represent all aspects of fungal presence in co-culture. To make sure our findings are consistent across these two experiments, we specify the difference in these two assays in the revised manuscript as the following: “*Since fungal spent media cannot fully recapitulate fungal presence in co-culture conditions, we tested whether fungal co-culture also conferred increased colistin survival.*”

Minor comments:

- For almost all the figures, blue and orange dots are used for 'monoculture' and 'coculture' respectively, while orange and black dots are used for WT and the mgtA mutant. However, the black and blue dots are hard to tell apart, and for several figure sub-panels, the legends are not provided (e.g. figures 2D, 2F, S9H), making it a little confusing to figure out what is being shown. It would be best if the WT and mgtA symbols were in colors completely different from the monoculture/co-culture colors, making it easier to tell those apart.
We have updated these figures as the reviewer suggested.

Line no 122 and Figure 1A. The term "defense genes" in bacteria typically refers to genes conferring protection against phage infections. Perhaps the authors can use a different term (e.g. 'protective genes').
We agree with the reviewer. We have changed “defense genes” to “fungal-defense genes” to disambiguate the terms.

Line no 186. 'However, neither MgtA...' should be 'However, neither MgtE...'
We thank the reviewer for pointing out this typo. We have fixed this in our revision.

Line no 268. Does fungal-mediated Mg2+ competition extend to Gram positive bacteria?
We thank the reviewer for raising this interesting point. MgtA is prevalent in diverse gram-negative bacteria but rare in gram-positive bacteria. Using the fitness effect of *mgtA* mutants in co-culture vs monoculture allowed us to infer Mg^2+^ competition easily for diverse gram-negative bacteria. Currently, we do not have the experimental tools to extend this finding to gram-positive bacteria. Co-culture growth kinetics for gram-positive bacteria are also likely to be different from gram-negative bacteria in a way that makes direct comparisons challenging. We have clarified our writing in the revised manuscript: “*This mode of competition might be highly specific between fungi and diverse gram-negative γ-proteobacteria we have tested…. Whether fungi can suppress gram-positive bacteria through the same mechanism of Mg^2+^ competition remains an open question*.”

Line no 314. It is unclear whether the 'transient co-culture' is the same or a different assay as the colistin survival assay.

We apologize for the confusion and have removed the word ‘transient’ for clarity. The assays is the same as the ‘colistin survival assay in fungal co-culture,’ where we co-cultured log-phase *P. aeruginosa* cells with *C. albicans* for 5 hours and treated them with colistin.

Line no 316. For the bacterial survival assays shown in figures 3 and 4 (and other supplementary figures), please provide absolute numbers as cfu (as in figures 1 and 2), as opposed to a percentage, for cell counts. This will allow readers to appropriately interpret the data.

We thank the reviewer for this suggestion. We now include the raw CFU counts of colistin survival assays in Fig 3 and 4 and other supplementary figures in new supplementary figures (Fig. S11, S13, S14, S15, and S17) in our revision.

Line no 934-5: Italicize P. aeruginosa.
This typo has been fixed in our revision.

Reviewer #1 (Significance (Required)):

This study identifies a novel interaction between two the co-infecting human pathogens Pseudomonas aeruginosa and Candida albicans, where C. albicans causes Mg2+ limitation for P. aeruginosa. Further, the authors show that this interaction affects levels of antibiotic resistance, as well as the adaptive mutations seen during the evolution of antibiotic resistance. This advances the field by delineating how microbial interactions can affect clinically relevant phenotypes, and potentially clinical outcomes. The study should be of interest to a broad audience of researchers studying microbial ecology, evolutionary biology, microbiology, and infectious diseases.

We are grateful for the reviewer’s positive appraisal.

**Reviewer #2** (Evidence, reproducibility and clarity (Required)):

This paper looks at the interaction between the fungus Candida albicans (Ca) and the bacterium Pseudomonas aeruginosa (Pa), which are found together in some environments. Co-culture experiments showed that Ca can inhibit the growth of Pa. The goal of this study is to determine the reason for this phenomenon and how widespread it is. This was performed by Tnseq analysis of Pa that identified 3 genes which showed significant decreases in the presence of Ca. Interestingly these were all in an operon that was recognized by the authors as being induced by RNAseq during co-culture. One of these genes, mgtA is a known Mg2+ transporter and therefore the remainder of the paper discusses the importance of competition for Mg2+.

The experiments seem to be well carried out and appropriately controlled.

We thank the reviewer’s appreciation of our science and the rigor of our experiments.

The use of the Mg2+ genetic sensor reporter in Pa is an interesting approach to determine the intracellular Mg2+ concentrations, however how these levels relate to one another between different experiments is not clear. In Fig. S5, the levels are 5 AU for growth in minimal media +low (10uM) Mg2+ and 38 AU for growth in minimal media+high (10mM) Mg2+. But the levels seen in Figure 1E are all much lower. With such low levels, it is difficult to determine if the impact of ∆PA4824 and ∆mgtA (while perhaps additive) are relevant. Would differences be seen with these various strains grown under different conditions?

We thank the reviewer for this query. The reviewer is right in that we do not use absolute quantification of intracellular Mg^2+^ levels. While our Mg^2+^ genetic sensor assay does not facilitate comparison of absolute Mg^2+^ levels across experiments, it provides a robust comparative measurement of relative intracellular Mg^2+^ levels in mutants versus WT cells, or between two different media conditions.

Using this Mg^2+^ genetic sensor assay, we tested intracellular Mg^2+^ levels of WT *P. aeruginosa* under various media conditions. We found that lower intracellular Mg^2+^ levels in *P. aeruginosa* cells and the requirement of *mgtA* in these media are well-correlated at lower total Mg^2+^ levels in media (Fig. S9A-E). In contrast, there are no significant differences in intracellular Mg^2+^ levels between Δ*mgtA* (or Δ*PA4824*) and WT cells in BHI media, which has higher total Mg^2+^ levels than fungal-spent BHI media. Our experiments reveal that the lack of *mgtA* or *PA4824* only affects intracellular Mg^2+^ levels when *P. aeruginosa* is cultured in media below a threshold level of Mg^2+^ concentration in media.

The experiments suggesting that the protein PA4824 is also a Mg2+ transporter seem to be related only to alpha fold predictions.

We clarify that our speculation that *PA4824* encodes a potential novel Mg^2+^ transporter was first motivated by finding that it is induced in low Mg^2+^ conditions, its genetic importance in Tn-seq experiments independent of mgtA, and our finding that cells with loss-of-function mutations in *PA4824* experience lower intracellular Mg^2+^ than WT cells. However, the reviewer is correct that this statement is speculative based on the Alphafold prediction. In the revised manuscript, we have clarified this point as the following: “*Based on our co-culture RNA-seq and Tn-seq experiments, results from the Mg^2+^ genetic sensor assay, and the Alphafold prediction of PA4824 protein structure, we speculate that PA4824 potentially acts as a novel Mg^2+^ transporter.*”

Is the statement in line 186 a typo? It is stated that "neither MgtA nor CorA was implicated in competition". Do the authors actually mean "MgtE"?

It is a typo. We thank the reviewer for pointing this out and have changed this to “MgtE”.

Reviewer #2 (Significance (Required)):

Ca and Pa are known to inhabit the same niches and previous studies have shown both can have antagonist effects on one another. Nutritional competition is one mechanism of antagonism that has not been that well studied between these two genera. That makes the finding of some significance and relevant to those with an interest in either of these microbes and co-infections. The authors also found that it was not just Ca that had this effect, but other fungi as well. And this effect was not just reserved to effect Pa, but also other bacteria, suggesting a more global impact.

We thank the reviewer for an accurate summary of our findings.

However, diminishing the impact of this finding is the question as to whether this is simply a phenomena seen under the very specific laboratory conditions tested here. Furthermore how these findings exactly relate to any infection environment is not clear.

Fungal-bacterial interactions occur in a variety of broad biological contexts, including during infection in animal hosts or in environmental-associated microbial communities. Our study is the first to identify nutritional competition for Mg^2+^ as one of the most important axes of competition between fungi and bacteria. Our study also identifies MgtA as one of the key bacterial genes that mediates this interaction. MgtA is only induced upon experiencing low Mg^2+^ conditions; the fact that most gram-negative bacteria encode MgtA implies they must encounter low Mg^2+^ conditions and face fitness consequences in those conditions. To address the reviewer’s concerns, we also highlight three additional points in our revised Discussion:

1. Fungal-bacterial competition for Mg^2+^ is not restricted only to BHI media alone. We also found the same phenomenon in TSB media medium. Indeed, we show (Fig. S9F) also that Mg^2+^ competition occurs whenever the environmental Mg^2+^ level is lower than 0.45mM, a critical threshold for fungi and bacteria to compete for this vital ion.
2. During infection in cystic fibrosis airways, proteomic experiments and Mg^2+^ measurement in CF sputum both suggest that *P. aeruginosa* experiences Mg^2+^ restriction.
3. Many previous studies have shown that many Gram-negative bacteria, including *Salmonella* Typhimurium, encounter reduced magnesium concentrations upon infection of hosts (PMID: 29118452). Our discovery that fungal co-culture may generally exacerbate fitness challenges associated with low magnesium levels is of high importance to all studies of gram-negative bacteria, not just to Pa.
4. In addition to infections in animal hosts, low Mg^2+^ is associated with worse outcomes of infections in plants. Our study suggests the importance of studying the role of Mg^2+^ competition in various infection contexts and the strategies of manipulating Mg^2+^ levels or fungal-bacterial interactions to constrain polymicrobial infectious diseases in diverse eukaryotic hosts and ecological conditions.

The authors also seem to vastly overinterpret the significance of their findings; the impact on Pa is only to slow growth, not necessarily effect fitness, per se. The final number of bacteria appears to be the same, it just takes slightly longer to get there.
We are puzzled by this comment from the reviewer; slow growth IS a fitness effect! Although we agree with the reviewer’s point that *C. albicans* is more likely to inhibit bacterial growth rate than viability (bacteriostatic, not bacteriocidal), there are many bacteriostatic antibiotic mechanisms.

In our co-culture assay, bacterial CFUs after 40 hours in co-culture are 10-100 times lower than in monoculture (this is not a subtle effect!). After 40 hours, bacterial cultures have already reached the stationary phase, which is why even slower growing bacterial cells in co-culture can ‘catch up’ (they are still lower by nearly 10-fold), despite fungal inhibition. Moreover, the co-culture condition provided enough of a fitness challenge to allow us to identify bacterial protective genes even in a pooled assay.

The authors speculate that that since Mg2+ supplementation did not totally restore growth to Pa during co-culture, that other Mg2+ independent "axes of antagonism" must exist. This also tends to diminish the significance of these finding.

Again, we are puzzled by this comment from the reviewer. Fungal-bacterial competition, like all microbial competition, is a multifactorial process, so we should not be surprised that Mg^2+^ isn’t the only axis of competition. Indeed, our study reinforces the importance of investigating all potential axes of competition to get a complete understanding of the mechanisms of fungal-bacterial competition.

The importance of mgtA on antibiotic susceptibility has been well studied in a number of bacteria including Pa making these findings generally confirmatory.

We would like to clarify this comment. To the best of our knowledge, *mgtA* in *P. aeruginosa* has not been reported in antibiotic susceptibility studies. Instead, *P. aeruginosa* *mgtE* is induced upon treatment with aminoglycoside antibiotics, but its expression does not change antibiotic resistance (PMID: 24162608).

The reviewer may be referring to studies in *S.* Typhimurium, where the Δ*mgtA* mutant shows increased susceptibility to nitrooxidative stress (PMID: 29118452) and to cyclohexane (PMID: 18487336), suggesting Mg^2+^ homeostasis might be generally important for bacterial survival to antimicrobial treatments. Although this is not the main focus of our study, we now include these references in our revised discussion to provide readers with more background on the relevance of our work: “*Mg^2+^ has been implicated in altering the susceptibility of gram-negative bacteria to antibiotics other than colistin. For instance, in S. Typhimurium, impaired mgtA or Mg^2+^ homeostasis increases susceptibility to cyclohexane or nitrooxidative stress. In line with these observations, our study also highlights the importance of studying how Mg^2+^ homeostasis broadly impacts antimicrobial resistance in gram-negative bacteria.”*

The importance of different mutations that emerge in Pa during mono vs. co-culture in the presence of colistin is not clearly explained. Why should co-culture inhibit the emergence of hypermutator Pa strains?

We thank the reviewer for the opportunity to clarify this important point. Previous studies have shown, both in Pa as well as other bacteria, that hypermutator strains often arise when bacteria adapt to strong and continuous antibiotic stress (PMID: 28630206) to maximize exploration of mutation space necessary to acquire beneficial resistance mutations even though hypermutation itself is inherently deleterious to bacterial fitness. We show that fungal co-culture protects *P. aeruginosa* from high concentrations of colistin by sequestering the Mg^2+^ co-factor required for colistin action (Fig. 4C). Thus, under co-culture conditions, bacteria experience lower levels of colistin than the levels administered and are subject to less severe fitness challenges, allowing them to eschew the deleterious route of acquiring adaptive mutations with hypermutation.

Our discovery that bacteria have an entirely different means of enhancing colistin resistance under fungal co-culture (or low Mg^2+^) conditions is one of the highlights of our study. Understanding the biological basis of this novel model of colistin resistance will be an active area of investigation to pursue in the future.

No additional experiments are likely needed but the authors should be encouraged to place their findings more clearly in what is already known in the field as well as articulate the limitations of their study.

We thank the reviewer for their detailed comments and suggestions. We hope our revisions have both clarified the importance and limitations of our study and provided the right context sought by the reviewer.

**Reviewer #3** (Evidence, reproducibility and clarity (Required)):

In this study, Hsieh et al. find a critical axis of competition between Pseudomonas aeruginosa and Candida albicans is Mg2+ sequestered by Candida. The authors find that use of BHI, which is has lower Mg2+ levels compared to other media, allowed this discovery. The authors further demonstrate critical genes for this axis in multiple gammaproteobacteria and fungal species. The authors further show that fungal Mg2+ sequestration promotes polymyxin resistance in multiple gammaproteobacteria and show that it alters the course of Pseudomonas aeruginosa evolution of polymyxin resistance. Finally, they show that for populations evolved polymyxin resistance in the presence of Candida, removal of Candida by antifungal treatment re-induces sensitivity to polymyxins.
We thank the reviewer for a concise and accurate summary of our study.

Major comments:
-The claims and conclusions are generally supported; however, a key phenotype of the ∆mgtA and ∆PA4824 mutants should be complemented in trans or in a second site of the chromosome.

We thank the reviewer for this comment and agree with the suggestion. In our revised manuscript, we now provide results of new complementation experiments recommended by the reviewer, which find that expression of *PA4824* or *mgtA* *in trans* restore the fitness cost of either deletion mutant (Fig. S4C and S4D).

-The authors note that "This mode of competition appears to be highly specific between fungi and gram-negative bacteria." However, it does not appear that gram-positive bacteria were tested in competition with fungi. Additionally, the only gram-negative tested were gammaproteobacteria (although do represent diverse gammaproteobacteria). This could be addressed by clarifying the text or OPTIONAL additional experimentation.

We agree with the reviewer. We had intended to highlight that we had only tested this mode of competition between fungi and gram-negative bacteria, but inadvertently phrased this to suggest that gram-positive bacteria are not subject to this competition. As we highlight in our response to Reviewer 1, we are unable to test this (so far) for gram-positive bacteria. We clarify this in our revision: ““*This mode of competition might be highly specific between fungi and diverse gram-negative γ-proteobacteria we have tested…. Whether fungi can suppress gram-positive bacteria through the same mechanism of Mg^2+^ competition remains an open question*.”

-Figure 3A: is this depiction of modifications on the O-antigen correct? PhoQ- and PmrB-activated enzymes seem to modify the lipid A portion of LPS (eg PMID: 31142822)

We thank the reviewer for noting this error, which we have now fixed in the revision.

- For many of the figures, multiple t-tests are used and it seems like perhaps an ANOVA with multiple comparisons would be more appropriate

We thank the reviewer for this feedback. In our revision, we now use Dunnett’s one-way ANOVA test for figures with multiple comparisons; our conclusions are unchanged.

Minor comments:
- The text and figures are clear and accurate

We thank the reviewer for this feedback.

-the cited nutritional immunity reviews are out of date (e.g. reference 37) and there are more recent reviews on the topic (e.g. PMID: 35641670)

We have added the suggested reference in our revision.

-Line 293: Unclear why polymyxin resistance would be "unexpected" following the explanation of why Mg2+ depletion might confer it

We agree and have removed ‘unexpected.’

-Line 318: "antibitoics" typo

We thank the reviewer for pointing out this typo, which we have now corrected.

Reviewer #3 (Significance (Required)):

The following aspects are important:

- General assessment: This study is very mechanistic, identifying the role of Mg2+ sequestration by fungi that limit gram-negative bacterial growth in Mg2+ deplete environments. The strengths are that relevant Mg2+ acquisition genes are identified or tested in Pseudomonas aeruginosa, the main test organism, as well as Salmonella enterica and Escherichia coli. Additionally, the authors identify a relevant Mg2+ mechanism in fungal species tested, including showing the importance with a genetic knockout. The limitations are relatively minor, and include lack of complementation, potential issues in model figure depiction of LPS modifications, and potential minor issues in statistical tests used. Future directions discussed include expanding analysis to clinical isolates, which is outside the scope of this manuscript which already showed the same mechanism in diverse gammaproteobacterial.
We thank the reviewer for their positive appraisal.

- Advance: This study has two major advances: The first is uncovering this critical Mg2+ sequestration axis in competition between fungal species and gammaproteobacteria. The second is the finding that the Mg+ sequestration induces polymyxin resistance and alters the evolutionary path to further polymyxin resistance. While nutrient metals as an axis of competition is not a conceptual advance, the specific role of Mg2+ and its affect on evolution of polymyxin antibiotic resistance is a conceptual advance.
- Audience: I think this study would be of interest to a relatively broad audience. The study itself touches on multiple fields including intermicrobial competition, nutritional immunity, antimicrobial resistance, and microbial evolution. Additionally, there are clinical implications for the potential to use antifungals to resensitize polymyxin-resistant P. aeruginosa to polymyxins.
- My field of expertise is bacterial genetics and physiology, nutritional immunity, and bacterial cell envelope. I do not have expertise in fungus.

We appreciate the reviewer’s positive and constructive feedback on our study and for highlighting the relevance of our research to a broader audience in microbiology and evolution. We do hope our mechanistic understanding of fungal-bacterial competition will spark further conversation or collaboration between evolutionary microbiologists and physician-scientists.
