# Supplementary material for: Widespread fungal–bacterial competition for magnesium lowers bacterial susceptibility to polymyxin antibiotics
Source: PLoS Biol. 2024 Jun 20;22(6):e3002694. doi: 10.1371/journal.pbio.3002694 (PMC11218974; doi:10.1371/journal.pbio.3002694)
Supplement: Attachment — Submitted filename: 20240508_response_to_reviewer_comments_v3YPH.docx [file pbio.3002694.s046.docx]

**Overall Comments**

We have made the following significant changes to the manuscript:

1. We have updated Fig 3A and Figure 5 based on the reviewer’s suggestions.
2. We clarified our text based on reviewer 2’s comments.

All changes in response to the reviewer’s comments are detailed in our point-by-point response (below).

**REVIEWERS’ COMMENTS**

**Reviewer #1**
**The authors have satisfactorily answered all the comments from the previous round of review. I have no further concerns.**
We thank the reviewer for their positive appraisal.

**Reviewer #2
This paper looks at the interaction between two co-infecting human pathogens Pseudomonas aeruginosa and Candida albicans. The authors found that C. albicans causes Mg2+ limitation for P. aeruginosa and go on to show the impact of this on P. aeruginosa growth and resistance to the antibiotic colistin. For the most part the experiments are well carried out but in a number places more explanation would seem to be needed:**

We thank the reviewer for their positive appraisal and we have made additional changes in response to their remaining concerns.

**-Title: "Antibiotic suscepibilty" would seem to be only for polymxyin-like antibiotics, so this should be rewritten to state this. Otherwise it implies that this is true for ALL antibiotics, which does not seem to be the case. Were other antibiotics tested?**

We have changed our title to “Widespread fungal-bacterial competition for magnesium lowers bacterial susceptibility to *polymyxin* antibiotics.” We point out in the paper that these are often last-resort use antibiotics and so quite important from a clinical perspective.

**-One Sentence Summary and line 339 and line 413: This sounds like the antibiotic is surviving. And this brings up a point, is the activity of colistin dependent on the level the Mg2+ (rather than an effect on Gram negative bacteria)? Has this been tested?**

We have changed line 339 to read “Fungal co-culture fundamentally alters the evolution of bacterial colistin resistance” and line 413 to “low Mg^2+^ was previously shown to affect colistin resistance in gram-negative bacteria.”

The pmrB experiments allow us to distinguish between the two possibilities suggested by the reviewer. Low Mg2+ conditions trigger *pmrB*-dependent changes in bacteria that enable them to overcome colistin. However, previous experiments have shown (and we confirm in the paper) that upon loss of *pmrB*, bacteria are still susceptible to colistin even in low Mg2+ conditions; this implies that it is the bacterial susceptibility rather than intrinsic colistin activity that is dependent on Mg2+ conditions.

**-Abstract and Figure 5 legend: What is non-canonical about this resistance? The low Mg2+ mechanism has been shown previously for P. aeruginosa and Salmonella typhimurium.**

The reviewer is correct that colistin resistance under low Mg^2+^ has been shown previously.

However, we are referring to the **mode of acquiring increased colistin resistance that is non-canonical**. Our evolution experiments show that Mg^2+^ depletion by *C. albicans* significantly alters how bacteria evolve colistin resistance in the long term. Our results clearly reveal that bacteria evolved in co-culture rely on a nearly mutually exclusive set of mutations from those acquired in mono-culture evolution to become colistin-resistant. Further, unlike mono-culture, co-culture-evolved resistance remains dependent on either fungal presence or low Mg^2+^ levels this phenotype has not been seen in our monoculture-evolved populations. As the other reviewers have previously pointed out, this result is unique in contrast to previous reports on colistin resistance in *P. aeruginosa* and other gram-negative bacteria. We have slightly changed the writing in the abstract to reflect this distinction.

**-Line 148: Is "~10% lower than WT" really statistically or biologically significant? On the other hand, the increased CFUs of monocultures of mutants compared to WT in Figure 1D are not mentioned.**

10% fitness reduction in a microbial population is quite biologically significant. It would take ~10 generations for the mutant population to go nearly extinct (which would occur within 6 hours in bacteria such as *P. aeruginosa*).

We did not observe a statistically significant difference in CFU between WT and mutants in Fig. 1D (blue dots).

**-Line 391: Is there a reference for the statement about the concentration of Mg2+ during P. aeruginosa infections in humans? Is this all infections or specific ones? If so, why is colistin ever effective?**

We included references for Mg^2+^ levels during infections in CF in line 391 as clinical studies in CF are relatively well-documented compared to other chronic infections.

[64] Cowley, E.S., et al., *Pediatric Cystic Fibrosis Sputum Can Be Chemically Dynamic, Anoxic, and Extremely Reduced Due to Hydrogen Sulfide Formation.* mBio, 2015. **6**(4): p. e00767

Colistin has been used as a last-resort antibiotic to treat multi-drug-resistant bacteria, but colistin treatment is not always effective. It is very concerning that the spread of colistin-resistant *P. aeruginosa* has been found in cystic fibrosis patients (https://doi.org/10.1016/j.jcf.2008.02.003, <https://doi.org/10.3109/00365548.2010.509333>).

**Figure 5: The designations in this figure are confusing (at least to this reviewer). Why is the symbol "|----" used? Maybe it would be helpful to have a cartoon of what the microbe looks like after colistin treatment. Also, does this imply the mechanisms for resistance to "high" or "low" colistin are different? This should be clarified**.

We thank the reviewer for this suggestion. We have updated Fig 5 accordingly.

**Reviewer #3**
**Generally, my comments are fully addressed.**
We thank the reviewer for their positive appraisal.

**I recommend additional minor revisions to the model figure in Figure 5A. The changes to Figure 5A for the representation of PmrAB- and PhoPQ-mediated resistance are good. (i) In figure 5A top left, I am confused as to why the Mg2+ and colistin are drawn only in association with the O-antigen portion, it is my understanding the Mg2+ association with LPS is critical for stabilizing the lipid A and inner core portions which have phosphate groups. For example, Mg2+ stabilization of the envelope is still important for organisms that do not have O-antigen such as Acinetobacter spp. and Neisseria spp. (ii) Similarly, the colistin mechanism of action includes incorporating into the lipid A portion of the outer membrane, not just associating with the O-antigen. (iii) I am also confused why it appears the O-antigen is separating from the lipid A, is this intentional? (iv) Finally, it looks like the authors have represented the outer membrane as comprised primarily of phospholipids with only a couple LPS molecules shown per area, which is not accurate to what we understand about the gram-negative OM; the outer leaflet should be predominantly LPS at least during homeostasis. These are minor issues but I think it is helpful to readers to draw the structures as accurately to what we know as possible.**
We thank the reviewer for these helpful suggestions (based on the description of the figure, we believe the reviewer is referring to Fig 3A). We have updated the figure as the reviewer suggested. In addition, we noted we mislabeled phoP and phoQ in the diagram, and we have corrected the error in this revision.

**Overall, this is an exciting, rigorous, and well-written manuscript.**

We thank the reviewer for their continued support of our study.
